# Sequences up- and down-stream of the DIS hairpin are important for HIV-1 replication

**DOI:** 10.1186/1742-4690-8-S2-P68

**Published:** 2011-10-03

**Authors:** Nikki van Bel, Atze T Das, Ben Berkhout

**Affiliations:** 1 Laboratory of Experimental Virology, Department of Medical Microbiology, Center for Infection and Immunity Amsterdam (CINIMA), Academic Medical Center of the University of Amsterdam, The Netherlands

## Background

Infectious HIV-1 virions contain two copies of the viral RNA genome, which are non-covalently linked through sequence elements in the 5'untranslated leader region (5'UTR). This leader region is highly structured and can expose the Dimerization Initiation Signal (DIS) in a stem-loop structure. Because of the palindromic nature of the hairpin loop, a kissing-loop dimer (KLD) interaction between two DIS elements can initiate dimer formation in vitro and subsequent RNA rearrangements can result in a more stable extended dimer (ED). Unpublished findings from our laboratory suggested that the unpaired nucleotides that flank the DIS stem-loop element may have a role in HIV-1 dimer formation. We therefore probed the function of these sequences during HIV-1 replication.

## Materials and methods

The sequences immediately up- and down-stream of the DIS hairpin were either mutated or randomized in the context of the HIV-1 molecular clone pLAI. The virus libraries with randomized sequences were cultured for several months to select for replication-competent variants. The effect of the mutations on viral gene expression, dimer formation, packaging and replication was analyzed.

## Results

We first determined the sequence constraints for the nucleotides flanking the DIS hairpin to support optimal virus replication. For this, we randomized these sequences and started multiple long-term cultures to select for replication-competent variants. This analysis revealed a strong preference for the wild-type sequence, but with some minor variations. Overall, these segments seem to play an important role in virus replication in a sequence-specific manner. Mutation of these sequences did not affect HIV-1 gene expression, but reduced viral replication. Further analyses of the precise replication step affected are ongoing.

## Conclusions

The single-stranded nucleotides flanking the HIV-1 DIS hairpin are important for efficient HIV-1 replication. Their role in viral RNA dimerization will be studied in further detail.

